# The Families of Non-LTR Transposable Elements within Neritimorpha and Other Gastropoda

**DOI:** 10.3390/genes15060783

**Published:** 2024-06-14

**Authors:** Donald James Colgan

**Affiliations:** Malacology, AMRI, The Australian Museum, 1 William St., Sydney 2010, Australia; don.colgan@australian.museum

**Keywords:** Gastropoda, Ingi, Jockey, Kiri, Nimbus, reverse transcriptase domain

## Abstract

Repeated sequences, especially transposable elements (TEs), are known to be abundant in some members of the important invertebrate class Gastropoda. TEs that do not have long terminal repeated sequences (non-LTR TEs) are frequently the most abundant type but have not been well characterised in any gastropod. Despite this, sequences in draft gastropod genomes are often described as non-LTR TEs, but without identification to family type. This study was conducted to characterise non-LTR TEs in neritimorph snails, using genomic skimming surveys of three species and the recently published draft genome of *Theodoxus fluviatilis*. Multiple families of non-LTR TEs from the I, Jockey, L1, R2 and RTE superfamilies were found, although there were notably few representatives of the first of these, which is nevertheless abundant in other Gastropoda. Phylogenetic analyses of amino acid sequences of the reverse transcriptase domain from the elements ORF2 regions found considerable interspersion of representatives of the four neritimorph taxa within non-LTR families and sub-families. In contrast, phylogenetic analyses of sequences from the elements’ ORF1 region resolved the representatives from individual species as monophyletic. However, using either region, members of the two species of the Neritidae were closely related, suggesting their potential for investigation of phyletic evolution at the family level.

## 1. Introduction

Transposable elements (TEs) are genetic elements with the ability to mobilise and replicate themselves in a genome [1]. They may comprise very large fractions of the genome and are being increasingly recognised as major influences on genomic evolution and biological and biochemical diversity in both vertebrates [2] and invertebrates [3]. Although there are broad patterns in TE distribution, such as a generally higher diversity in fish than in birds and mammals [4], there are considerable differences within phyla. For example, the TE content of fish ranges from 5% to 56% of the genome [5]. TEs can comprise large fractions of gastropod genomes [6,7,8] However, despite the ecological importance of Gastropoda and their great biodiversity, knowledge of their complement of such elements is only now beginning to accumulate.

Most targeted investigations of TEs including gastropod taxa have focussed on one of the five major groups of retrotransposons [9]. These are (1) short interspersed nuclear elements (SINEs), which were investigated by Luchetti et al. [10] and Matetovici et al. [11]; (2) DIRS1, which is represented by a few copies in the genomes of *Aplysia californica* (Cooper, 1863) and *Lottia gigantea* Sowerby I, 1834 [12]; (3) long terminal repeat (LTR) transposons, which were investigated in detail by Thomas-Bulle et al. [13] who reported that the majority of such elements belong to Gypsy-like clades, with Copia-like elements found at low frequency, and BEL/Pao elements being found only in *A. californica* (at low frequency) and in *L. gigantea* (abundantly); (4) retrotransposons that do not end with long terminal repeats (non-LTR) of which one type, Nimbus, was investigated in *Biomphalaria glabrata* (Say, 1818) by Raghavan et al. [14]; and (5) Penelope-like elements which reportedly occur in gastropods [6,8], although in the latter article they are treated as a SINE, and there has been no detailed characterisation of the group’s representatives in these molluscs.

The genetic structure of non-LTR transposons usually includes two open-reading frames (ORFs). ORF1 contains sequences which have RNA-binding and nucleic acid chaperone activities and may play a similar role to the gag proteins of retroviruses [15], although these sequences are not phylogenetically closely related [16]. ORF-2 has three principal domains. The first of these (near the 5′ end of the ORF) is frequently designated as the “Exonuclease-Endonuclease-Phosphatase” (EEP) domain or the “Apurinic Endonuclease” domain. The second domain is the reverse transcriptase and the third (towards the 3′ end of the ORF) is the non-LTR RNase HI domain of reverse transcriptase [15,17].

There are numerous families of non-LTR transposons, 33 main types being currently listed in Repbase https://www.girinst.org/repbase/update/browse.php (accessed on 4 June 2024 [18,19]. These are classified in five superfamilies (I, Jockey, L1, R2 and RTE) [17]. Individual copies of the transposons or fragments of them have been usually classified on the basis of the protein sequence of the RT domain [20]. 

TEs, including non-LTR TEs, are mobile within the genomes of individual species and also between species. Transposition of non-LTR TEs is mediated by transcription products of the element itself [16]. Whether because of host processes to inactivate TEs [21,22], competition from other TEs (such as insertion) [22] or other types of inactivation, particular copies of TEs may become incapable of transposition. Transmission may be of partial elements, possibly including entire ORFs [17]. It is known to occur at considerable frequency in *Drosophila* [23] as assessed by three main criteria—phylogenetic incongruence between the TE tree and the host tree; an irregular distribution of the element in a group of species; and high similarity between sequences of TEs from distantly related species. 

The non-LTR elements have not been the subject of a targeted investigation in Gastropoda although they may comprise as much as one third of the genome, e.g., in *Cepaea nemoralis* (Linnaeus, 1758) [8]. Despite this, sequences in assemblies of NGS datasets from Gastropoda are now frequently described as non-LTR transposons without being characterised in sufficient detail to be identified as belonging to particular types. For example, keyword searching of the GenBank nr database found no references to the families RTEX and Ingi and only one accession referring to Nimbus, RTE-like and CR1 [14]. 

This investigation was conducted to characterise non-LTR transposons in Neritimorpha, with a consideration of their distribution in the other main lineages of gastropods. It was prompted by the suggestion that TEs occur in genomic skimming surveys from neritimorph species [24] prepared for use in mtDNA investigations for phylogenetic analyses [25] and characterisation of aquaporin proteins [26]. Many sequences in these surveys were described in Blast2GO analyses [27] as similar to the reverse transcriptase of the TE Jockey, first identified in *Drosophila melanogaster* [28,29], although this particular element was supposed not to occur in Mollusca [17,20].

Neritimorpha is a relatively small group in terms of the number of species [30] but forms one of the five major gastropod evolutionary lineages [31,32] and inhabits a great variety of environments. It has four extant superfamilies [33], two of which, Helicinoidea and Hydrocenoidea, are predominantly terrestrial. The four other major lineages in Gastropoda are the Patellogastropoda, Vetigastripoda, Caenogastropoda and Heterobranchia, the two latter comprising the Apogastropoda and which together contain the vast majority of species in the class. 

The investigation was based on genomic skimming surveys of three species, *Pleuropoma jana* (Cox, 1872) (Helicinoidea: Helicinidae), *Georissa laseroni* (Iredale, 1937) (Hydrocenoidea: Hydrocenidae) and *Nerita melanotragus* (Smith, 1884) of the predominantly marine family Neritidae, and the recently published draft genome of *Theodoxus fluviatilis* (Linnaeus, 1758) [34] which is also a member of the Neritidae but inhabits freshwaters. The aims of the investigation were to establish the presence of non-LTR TE families in the studied species, to provide an initial estimate of their relative importance in neritimorph genomes, and to examine the phylogenetic relationships of any representatives that might be found. Both ORF1 and ORF2 were investigated, although attention was focussed on the reverse transcriptase domain of the latter.

## 2. Materials and Methods

### 2.1. Neritimorph Genome Skimming Data

Details of the collection of the genome skimming data from individual snails of three neritimorph species are provided in [26]. Contigs were assembled from the raw reads after trimming using default parameters in CLC Genomics Workbench (http://www.clcbio.com/products/clc-genomics-workbench/ accessed 26 November 2019), after, using a word length of 20, bubble size of 30 and the “fast” algorithm. 

The snail specimens used were AMS C.553331 (*N. melanotragus*), AMS C.553328 (*P. jana*) and AMS C.553329 (*G. laseroni*). The individual sequence reads are available as a GenBank Short Read Archive (SRA) submission: BioProject ID PRJNA481126 with the run identifications: SRR7611487 for *P. jana*, SRR7609217 for *N. melanotragus* and SRR7522894 for *G. laseroni*. Contigs in the assemblies are designated below by the initials of the species binomen and a number. Potential coding sequences within a contig are identified by the number allocated during the “getorf” analyses described below.

### 2.2. Analytical Procedures

Analyses using the Galaxy platform [35] were conducted on the Galaxy Australia webservers at the University of Queensland (https://usegalaxy.org.au/ accessed 8 June 2024). The NCBI BLAST+ programs blastn, tblastn, tblastx and blastx were used to search databases prepared by the program makedb from fasta format DNA or protein sequence data sets [36,37]. Repeatmodeler [38] was used for preliminary invstigations of the repeat sequences in the genome skims. The EMBOSS getorf program was used to search for potential open reading frames sequences [39]. The settings used in analyses with this program were to search both strands for sequences between stop codons, sequences to have a minimum length of 100 bases (DNA) and not to requires all start codons to code for methionine. The program “Select sequences by ID” [40] was used to select subsets of sequences from fasta format files and the program “Seqkit” to order sequences in such files [41].

BioEdit [42] was used for visual examination of data and for preparing subsets of the alignment positions or taxa. Clustal X2 [43] was used for multiple sequence alignment with default parameters.

Maximum likelihood (ML) phylogenetic analyses of amino acid sequences were performed on the CIPRES data portal [44]) using the RAxML Blackbox [45] with default assumptions (not using empirical data frequencies, no invariable sites). The required number of rapid bootstrap replicates was calculated by the majority rules extended (“MRE”) bootstopping criterion [46]. The LG substitution matrix [47] was used for analyses of amino acid sequences. Phylogenetic trees were examined using Figtree v. 1.4.2. [48] which was also used to output graphics files for topology illustration. Sequences found to be highly divergent in preliminary analyses were checked by blastp searches of GenBank and were removed from the dataset if found to derive from contaminating micro-organisms. 

### 2.3. Dataset Compilation for ORF1

Sequences of the Jockey element of *D. melanogaster* were used to initiate databases searches for non-LTR TEs because this is a canonical example of TEs in invertebrates and is very well characterised structurally. The “nucleic acid binding protein” sequence (GenBank accession AAA28939) was used to search neritimorph genomic skimming sequences. The sequence from each neritimorph species with the longest homologous alignment) to AAA28939 (and in each case the lowest E-value) was used to identify conspecific contigs with similar sequences by tblastx (E < −7). The longest protein sequences found for each contig using getorf were combined and aligned using default parameters in ClustalX2 [41] for preliminary phylogenetic analyses. 

Representative sequences from the neritimorph alignment were used to find similar sequences in the GenBank nr database with blastp (E < 0.001). The scaffolds for the genome of *T. fluviatilis* [34] available in the GenBank WGS database were searched with tblastn (default parameters) using Gl contig 19576 ORF 45 GenBank sequences and all neritimorph sequences were combined into a single file and re-aligned with Clustal X2. The alignment is appended to this article as supplementary file “ORF1.fasta”.

### 2.4. Dataset Compilation for ORF2

The *D. melanogaster* protein sequence for RT-pol (GenBank accession AAA28675) was used to search neritimorph databases with tblastn, and an expect value upper limit (E < −6). The longest amino acid sequences from each neritimorph species with similarity to the *D. melanogaster* protein (G29_1719, Nm_685 and H2_16092) were each used in tblastn searches of all three neritimorph databases. After filtering to remove duplicates and all sequences less than 230 amino acids in length, the data from the three species were combined. After alignment with Clustal X2, the dataset was trimmed to a region corresponding to positions 452-803 of the *D. melanogaster* GenBank accession AAA28675 (which includes the RT domain in positions 497-757). Then neritimorph sequences with less than 67% coverage of this region were removed. 

Representative sequences from the main clades in phylogenetic analysis of the neritimorph ORF2 dataset were selected to be used as “classification sequences”. These were used to assist in the identification of non-LTR family types and database searches.

 Non-LTR TE sequences in *T. fluviatilis* from the GenBank WGS database were sought by tblasn queries with each classification sequence, limiting the number of retrieved accessions to 1000. The complete scaffolds were downloaded and combined into a single fasta file from which duplicates were removed. This was uploaded to Galaxy and analysed by getorf, setting the length of proposed ORFs to be greater than 300. The resulting ORFs were searched with all classification sequences using blastp and E < −5. ORfs meeting these criteria were added to the neritimorph ORF2 dataset and re-aligned with Clustal X2. The alignment is appended to this article as supplementary file “ ORF2_Neritimorpha.fasta”.

### 2.5. Element Class Presence

The ORF2 classification sequences were used as queries in RTclass1 (https://www.girinst.org/RTphylogeny/RTclass1/ accessed on 4 June 2024) [20] to determine the retrotransposon family in the Repbase [18,19] database of repeated sequences to which they are most phylogenetically similar. 

The classification sequences were then used in tblastn searches of the neritimorph databases (E < −7) to estimate the numbers of copies of particular element types each contained. When calculating this, the estimate was based on the numbers of distinct contigs with length greater than half of the alignment length thus ensuring that the counts did not include two target contigs (disjunct but genomically adjacent) corresponding to the same search contig. Classification sequences were individually used to search GenBank (E < −5). Additional blastp searches for Ingi sequences were conducted using the GenBank accession GFR70456.1 from *Elysia marginata* (Pease, 1871) as this element was not initially found in the neritimorph data. One search was limited to heterobranchs (E < −11, limited to 1000 sequences) and another to non-heterobranch Gastropoda (E < −06). The results of GenBank searches were combined and individual sequences allocated to the category of the best scoring (E-value) classification sequence.

### 2.6. Variability along the ORF2 Sequence

An investigation of patterns of variability along the ORF2 sequences was made using the program MEGA11 [49] to estimate position by position substitution rates in amino acid sequence alignments using the topology from the ORF2 analysis below as a user tree.

## 3. Results

The approximate total lengths of the contigs in the genomic skims (Supplementary Table S1) were 0.245 Gb for *G. laseroni*, 0.555 Gb for *N. melanotragus* and 0.363 for *P. jana*. These can be compared to the estimated sizes of the complete genomes of other Neritidae [50] which have been reported as 1.382 Gb for *N. albicilla* Linnaeus, 1758; 1.422 Gb for *N. helicoides* Reeve, 1855, 1.921 Gb for *N. japonica* Dunker, 1860, 2.191 Gb for *Vittina plumbea* (Sowerby II, 1849) and 1.449, 1.562 and 1.791 Gb respectively for three species of *Clithon* Montfort, 1810. The comparisons suggest that >15% of each neritimorph genome has been included in the skimmed sample.

### 3.1. Phylogeetic Analysis of ORF1 Sequences

The ln likelihood of the ML tree for the ORF1 dataset was −51222.922. The number of bootstrap replicates determined by the MRE criterion was 550.

**Figure 1:**
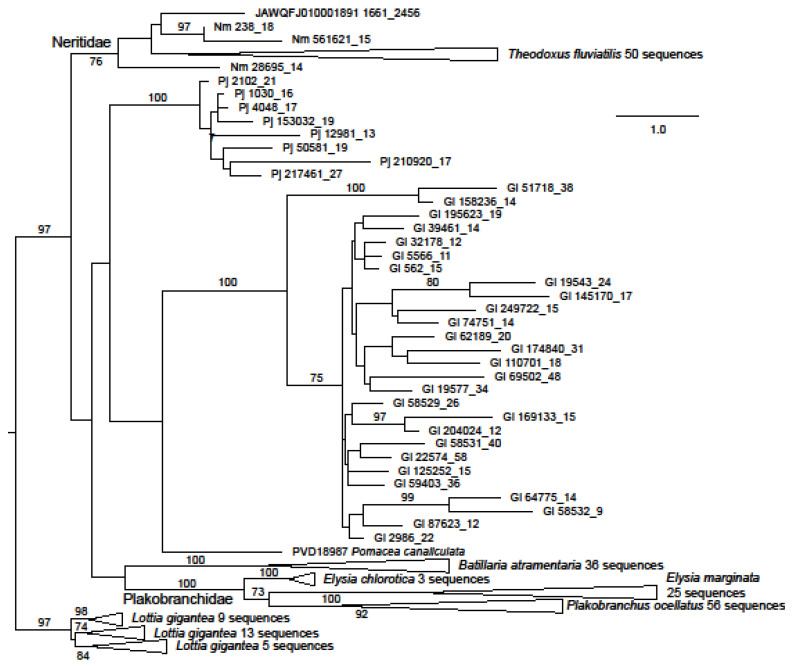
Maximum likelihood phylogeny of ORF1 in the Neritimorpha and other Gastropoda. For GenBank data. the source of individual sequences is indicated by accession numbers and species name or the scaffold number in the *T. fluviatilis* genome followed by the contig number and the relevant ORF from the contig. The source for sequences from the genomic skimming surveys is indicated by Gl for *Georissa laseroni*, Pj for *Pleuropoma jana* or Nm for *Nerita melanotragus.* These sequences are identified by contig numbers followed by the ORF number from the contig. The sequences from Patellogastropoda are used as an outgroup. The triangles represent multiple sequences from the indicated species. Bootstrap values above 70% are shown along branches. The scale bar represents estimated substitutions per site.

The great majority of identified neritimorph ORF1 coding contigs were from *G. laseroni* or *T. fluviatilis* (Figure 1), 129 sequences being found in the latter species. Few sequences representing ORF1 were found in *P. jana* (eight sequences) or *N. melanotragus* (3). The sequences from *G. laseroni* and *P. jana* were both resolved as monophyletic with 100 percent bootstrap support, with those from the former being divided into two robustly supported clades, which were most closely related to the same sequences in GenBank in blastp searches. Two gastropod families were represented by multiple species in the ORF1 dataset. The sequences from each were monophyletic with bootstrap support of 100% for Plakobranchidae (two species of *Elysia* and *Plakobranchus ocellatus* van Hasselt, 1824) and 76 for Neritidae (*N. melanotragus* and *T. fluviatilis*). Sequences from Neritimorpha were shown as paraphyletic with respect to other gastropod groups (excepting Patellogastropoda) but without strong bootstrap support.

### 3.2. Analyses of ORF2 Sequences

#### 3.2.1. Phylogenetic Analysis of ORF2 Sequences

The final ORF2 alignment included 57 sequences from *G. laseroni*, 7 from *N. melanotragus* and 60 from *P. jana.* The low numbers of sequences from *N. melanotragus* is notable especially as this had the highest summed contig lengths of the three species (Supplementary Table S1). The discrepancy between species was not as marked when less stringent selection protocols were used. For example, tblastn searches using the classification sequences with an E value of −3 and no length restriction found 1785 unique contigs in *N. melanotragus* compared to 3216 in *G. laseroni* and 2012 in *P. jana.*

**Figure 2:**
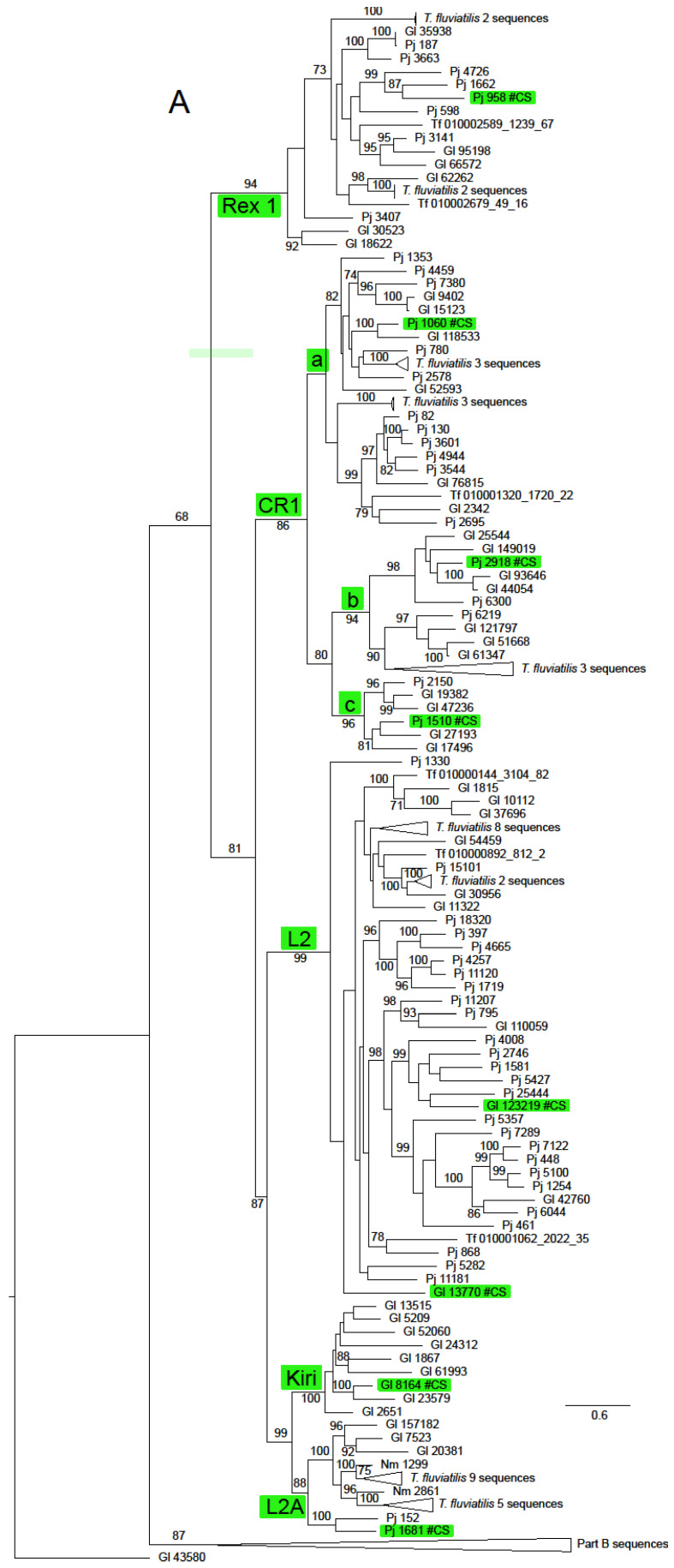

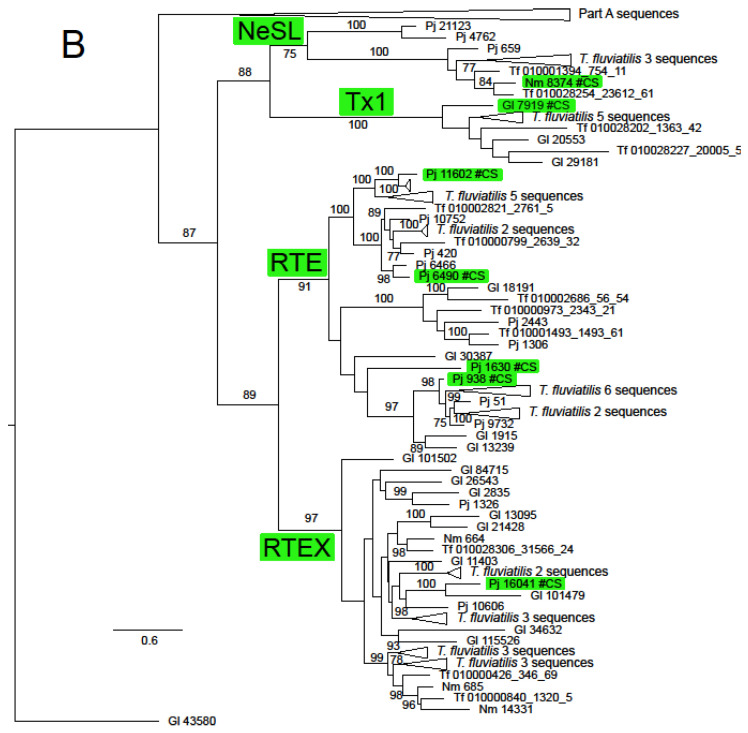
Maximum likelihood phylogeny of the RT domain and surrounding regions of ORF2 in Neritimorpha. (**A**) Part A of the topology; (**B**) Part B of the topology. Non-LTR families and classifycation sequences are highlighted inside green boxes. The source of individual sequences is indicated by Gl for *Georissa laseroin*, Pj for *Pleuropoma jana*, Nm for *N. melanotragus* or the letters Tf (replacing “JAWQFJ” in the scaffold numbers) for *T. fluviatilis.* Sequencea are identified by the contig numbers in the genomic skimming surveys or the scaffold number in the *T. fluviatilis* genome followed by the contig number and the relevant ORF from the contig. The topology is unrooted. The triangles represent multiple sequences. Most refer to sequences from *T. fluviatilis* but the bottom triangle in part A refers to the sequences in the part B topology and the top one in part B to the Part A sequences. Gl contig 43580 is shown in both parts for orientation. Bootstrap values above 70% are shown along branches. The scale bar represents estimated substitutions per site.

The *ln* likelihood of the ML tree for the ORF2 dataset was −228186.661. The number of bootstrap replicates determined by the MRE criterion was 400. There was considerable structure in this tree, with very strong bootstrap support for all of the non-LTR families (Figure 2 and Supplementary Figure S1). There was also support for some groupings of families or superfamilies, for example, 88% for the pairing of the superfamilies L1 (represented by Tx1) and R2 (represented by NeSL); the Jockey superfamily had bootstrap support of 68%. The families included in this group except Rex1 were resolved in a clade with support of 81%. The RTE superfamily (families RTE and RTEX) had support of 89%. The grouping of the superfamilies L1, Tx1 and RTE had support of 87%. The CR1 clade was notable for the number of multi-member, basal sub-clades, many of which were robustly supported. Some of these sub-clades had restricted taxonomic distributions that did not include sequences from Neritidae and others in which this family was represented by only a few *T. fluviatilis* sequences. 

Only two non-LTR families, RTEX and L2A, were found in all four neritimorph species. *N. melanotragus* sequences were not found in Tx1, RTE, Rex1, CR1, L2 or Kiri clades. Sequences from *P. jana* were not found in Tx1 or Kiri, those from *G. laseroni* not in NeSL and those from *T. fluviatilis* not in Kiri or CR!a, one of the sub-clades of CR1. Sequences from the different species within a family-level clade were mostly interspersed, with some notable exceptions. There was a strongly supported (bootstrap 99%) sub-clade of RTEX that contained only *N. melanotragus* and *T. fluviatilis* sequences and a sub-clade in L2A (not strongly supported) that contained sequences only from these species. Indeed all *N. melanotragus* sequences were resolved in robustly supported clades of which the other members all belonged to *T. fluviatilis.* All members of the Kiri lineage in this analysis belonged to *G. laseroni*.

The analysis of ORF2 including data from Neritimorpha and some other Gastropoda (alignment appended as supplementary file ”ORF2_Gastropoda.fasta” resulted in a topology) (Supplementary Figure S2) that was generally characterised by the occurrence of clades that did include considerable numbers of sequences from a particular species but which were not strongly bootstrap supported and which also included one or two sequences from the Heterobranchia, particularly *B. glabrata*. Two notable exceptions to this pattern were a large clade of sequences from this species and two sequences from *Elysia chlorotica* (Gould, 1870) (bootstrap support of 44%) and a clade containing the Nimbus classification sequence Nm contig 7015, two sequences from *G.laseroni*, 6 from *T. fluviatilis* and 17 from *P. ocellatus* (bootstrap support of 35%). 

#### 3.2.2. The Distribution of ORF2 Sequences in Gastropoda

The numbers of members of various superfamilies and families of non-LTR transposons found in the present investigation characterised by standard resemblance criteria using Repbase are detailed in Table 1. Subdivisions are recorded for CR1 showing the sequence numbers recovered by the three classification sequences from this family which is partitioned in this way because the lineages containing them were resolved into distinct clades in the neritimorph phylogenetic analysis (Figure 2A). At the family level, all were found in the *T. fluviatilis* draft genome and almost all were found in each of the three neritimorph genome skims, the exceptions being Nimbus in *P. jana* and Ingi, CR1 and L2 in *N. melanotragus*. The distribution of non-LTR families in other gastropods suggested that notable differences between groups might be found by more targeted searches. Considerable numbers of non-LTR TEs were found in the species *Batillaria atramentaria* (Sowerby II, 1855) (Caenogastropoda; Cerithioidea), *Pomacea canaliculata* (Lamarck, 1822) (Caenogastropoda: Architaenioglossa), *E. marginata* (Pease, 1871) and *P. ocellatus* (both Heterobranchia: Sacoglossa). Most families were found in *P. canaliculata*. These species all had large numbers of Ingi sequences, but all bar *P. canaliculata* had only a smattering of other families of non-LTR TEs except that *B. atramentaria* had 31 sequences from the Rex1 family. 

The distributions of non-LTR families considered in Table 1 were based on relatively stringent inclusion criteria. These were relaxed in the tblastn searches using the classification sequences with an E value of −3. In these all were represented by contigs in the genome skims to which they were more similar than any other classification sequence. The removal of the minimum length criterion (>230 positions) was largely responsible for this. 

A broader distribution was also suggested by the Repeatmodeler results, although these also revealed considerable differences between neritimorph species (Table 2). *Nerita melanotragus* had a relatively low number of “consensus sequences” from the Jockey superfamily (all from the CR1, L2 or Rex families) compared to the other two species and a much higher proportion of the RTE superfamily. Some sequences described as Penelope were found in these analyses but this identification was not confirmed by blast+ searches with known sequences of this element deposited in GenBank. There were additionally a few sequences (less than four in each species) that were described as “unkmown” LINE elements but these were apparently bacterial contaminants (blastp searches of GenBank).

#### 3.2.3. Variability along the ORF2 Sequence

**Figure 3:**
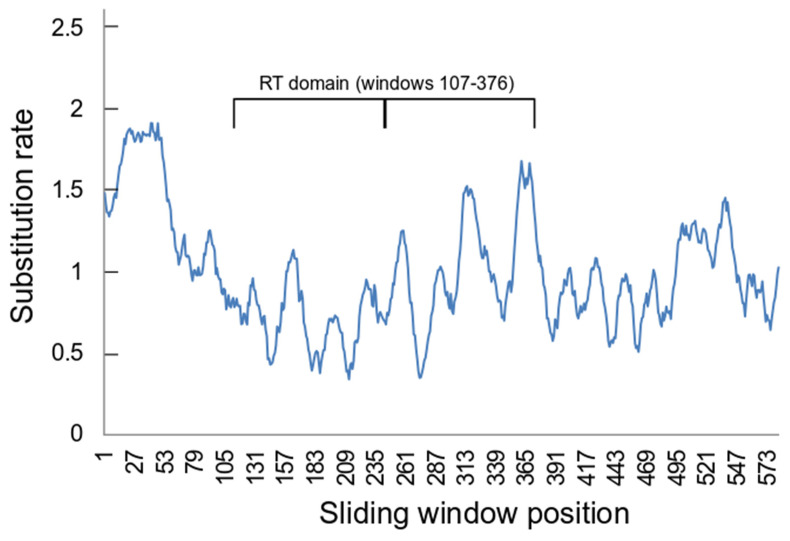
Sliding window estimates of position by position substitution rates. The estimates were calculated in MEGA 11 [49] for a sliding window of 20 positions with a minimum 5 sequences. The RT domain occupies sliding window numbers 107–376 (corresponding to positions 126–395 in the ORF2 alignment). The thick line in the RT domain span separates the two halves of this region.

The sliding window estimation of position by position substitution rates suggested that there were differences along the length of the aligned ORF2 sequences (Figure 3). For example, there were considerable differences in the average estimated substitution rates between positions which belong to the RT domain (0.866 ± 0.62), which corresponds to positions 126-395 in the ORF2 alignment, and those that do not (1.111 ± 0.619) (*p* << 0.001 by *t*-test assuming unequal variances). Notably there also significant differences between the two halves of the RT domain, comparing windows 107-241 (0.703 ± 0.546) with windows 142-376 (1.035 ± 0.647) (*p* << 0.001 by *t*-test assuming unequal variances).

## 4. Discussion

The investigations reported here revealed that there is a very broad taxonomic distribution of non-LTR TEs in Gastropoda, consistent with the patterns shown in other taxa. The distribution of families and superfamilies of the elements is not however uniform across the class and the patterns of their variation supports the suggestion that Gastropoda are a very good model to investigate the evolution of repeated sequences [13].

All five non-LTR superfamilies as defined by [17] were found in all of the four studied Nertitimorpha, represented by sequences that were identified using relatively stringent selection criteria. Most families of the non-LTR TEs found in any of the neritimorphs were also recorded in most of its studied species. However there were notable exceptions to this, particularly in the apparent absence of families from *N. melanotragus*, including some that were found in *T*. *fluviatilis* which belongs to the same gastropod family. However, the families found in any studied gastropod that were missing from individual neritimorph species were all found to be represented in them using less stringent identification criteria in tblastn or Repeatmodeler searches. The apparent presence or absence of a particular TE may therefore be confounded by the degree of differentiation of elements. It is notable, however that the reported absence of the Jockey family in Gastropods [17,23] is supported by its absence from the species studied here.

Some abundance differences were found between the non-LTR complement of the studied species. Ingi is very frequent in the heterobranch family Plakobranchidae but mostly absent from the neritimorphs except *T. fluviatilis*. Its abundance in this species suggests that its numbers can readily be expanded or contracted in different lineages within the same family. The CR1 superfamily of ORF2 sequences is very well elaborated in Neritimorpha and showed considerable phylogenetic structure within the group which may be useful for investigating questions about lineage assortment and reticulate evolution [15,22].

There was variation in estimated substitution rates along the neritimorph non-LTR TE alignment, notably between the two halves of the RT domain. One possible explanation of this is that some regions of ORF2 have higher rates of substitution when non-LTR elements are active than others, particularly the RT domain which is apparently evolutionarily conservative within this ORF [17]. The rates of substitution would be expected in general to be similar in all regions once the elements become inactive. However, observed similarity differences may reflect the substitutions that occurred before elements became inactive. If similar difference patterns were found in other taxa, they might be informative about the significance of the various structures revealed in fine-scale analysis of non-LTR TE RT domains [51].

The ORF1s of Mollusca are very divergent from other taxa as suggested in [17]. The patterns in the topology of the sequences from this region also differed from those in the ORF2 phylogenies, especially in that the sequences from *G. laseroni* and *P. jana* were monophyletic in the former but were very much interspersed in the latter with the exception of Kiri. Differences in evolutionary conservatism might be part of the explanation of this contrast between between ORF1 and ORF2 phylogenies. ORF1 is more conservative than ORF2 in intact elements although not in those that are incomplete [52]. The lineage sorting of incomplete elements that would be required to produce the ORF1 monophyly would also have occurred in ORF2 elements. Possibly, although this is speculative, the numbers of ORF1 sequences may be sufficiently lower than the numbers for ORF2 for the sorting required to produce monophyly to proceed more effectively in the former.

There was no evidence of transposition by horizontal transfer in the present results based on the criteria of Tambones et al. [23]. In particular, there was little phylogenetic incongruence between the TE trees and the host phylogeny for either ORF1 or ORF2. The distribution of elements within Neritimorpha was not notably sporadic and there were no instances of unexpectedly high similarity between TEs from distantly related species. It would be interesting to investigate whether the horizontal transposition of non-LTR TEs is uncommo in Nertitimorpha in more detailed studied. Horizontal transfer is rare in mammals [1] and the possibility has been generally contradicted for at least one element [53]. The spectrum of non-LTR family presence in more stringent ORF2 searches was more similar in *G. laseroni* and *P. jana* than between either of these and *N. melanotragus*. The latter as a member of a derived family in the phylogeny of Neritimorpha [25]. This, together with the finding that it has representatives of most non-LTR families in less stringent searches (but not in more stringent searches) suggests that there has been a general pattern of non-LTR TE sequence erosion in this species, rather than replenishment by horizontal transfer. 

This is the first research to make a focussed investigation of the presence and evolution of the non-LTR TEs in the important animal class Gastropoda. Its results suggest a number of areas that might be fruitful for understanding the evolution and biological function of non-LTR TEs. (1) In-depth investigations may be useful for phylogenetics, especially once sufficient information has been accumulated to allow for the effects of any horizontal transfer events that might have occurred during the evolutionary history of a group. The CR1 superfamily may be especially interesting in this context. (2) Detailed comparisons of the patterns of sequence variation in individual families of non-LTR TEs in particular groups of Gastropoda may illuminate their biological function. The observed differences along the RT domain are of particular interest here. Similar approaches have already been found to be useful, for example in metazoan-wide surveys of the R2 elements [53]. (3) The use of classification sequences in conjunction with Repbase may be a practical introduction to studies the non-LTR TEs of relatively understudied organisms, especially within families. This is needed because of the generally poor description of such sequences in GenBank databases and the current bottleneck in genome analyses that is caused by lack of the proper annotation of TEs [4].

## Figures and Tables

**Table 1:** Distribution of families of non-LTR transposons in gastropod speces according to the classification of reverse transcriptase types.

Source ^1^ Non-LTR Classification ^2^	Lg	Ga	HA	Gl	Pj	Nm	Tf	Pc	Ba	Ls	Ac	Po	Ec	Bg
**I superfamily**														
**Nimbus**		3		9		1	127	2			1	1		2
**Ingi ^3^**		59		11	2		249	41	273		1	247	65	17
**Jockey Superfamily**														
**CR1a**		1	1 Hdh	16	3		3	1		1				1
**CR1b**	1	1	1 Ht	55	18		215	2		1	3	1	1	1
**CR1c**	2	1	1 Hd	20	3		24	3		1	2			1
**Kiri**	2	4		58	2	12	121	1	1					
**L2**				66	34		520	6			2			1
**L2A**				14	9	7	138	15		2		1		
**Rex 1**				38	13	1	137	1	31		1	4	4	
**L1 Superfamily**														
**Tx1**	1	7	1 Hd	46	1	7	235	2		1	1	1		1
**R2 superfamily**														
**NeSL**				6	4	10	255				2			
**RTE superfamily**														
**RTEX**	2	4	1 Hr	95	7	31	538	6			1			
**RTE**	1	3		55	24	14	916	19		1	6			19

^1^ Lg: *Lottia gigantea*; Ga; *Gigantopelta aegis* Chen, Linse, Roterman, Copley & Rogers, 2015; HA: *Haliotis* Linnaeus, 1758; Hdh: *Haliotis discus hannai* Ino, 1953; Hd: *Haliotis diversicolor* Reeve, 1846; Hr: *Haliotis rufescens* Swainson, 1822; Ht: *Haliotis tuberculata* Linnaeus, 1758; Gl: *G.laseroni*; Pj: *P. jana*; Nm: *N. melanotragus*; Tf: *T. fluviatilis*; Ba: *Batillaria atramentaria* (Sowerby II, 1855); Pc: *Pomacea canaliculata* (Lamarck, 1822)*;* Ls: *Littorina saxatilis* (Olivi, 1792); Ac: *Aplysia californica*; Po: *Plakobranchus ocellatus;* Ec *Elysia chlorotica*; Bg: *Biomphalaria glabrata*. ^2^ The other species in which non-LTR TEs were found, as singletons unless specified, were *Patella vulgata* Linnaeus, 1758 L2; *Peringia ulvae* (Pennant, 1777) L2; *Crepidula coquimbensis* Brown & Olivares, 1996 L2; *Aliger gigas* (Linnaeus, 1758) RTE; *Rapana venosa* L2, L2a; *Tritia neritea* (Linnaeus, 1758) RTEX; *Elysia marginata* Rex1 3 sequences, Ingi 539 sequences; *Lymnaea stagnalis* (Linnaeus, 1758) L2; *Oxyloma* Gardner & Campbell, 2002—sp. HWH-2012 RTE 12 sequences; *Candidula unifasciata* (Poiret, 1801) Ingi 9 sequences. ^3^ Searched with GFR70456 using blastp as Ingi was not initially found in Neritimorpha.

**Table 2:** The number of consensus sequences in non-LTR TE superfamilies found in neritimorph species by Repeatmodeler. The species designations are Gl for *G.laseroni*. Nm for *N. melanotragus* and Pj for *P. jana*.

Species TE Superfamily	Gl	Nm	Pj
I ^1^	27	3	19
Jockey	169	125	216
L1	16	29	4
R2	12	46	9
RTE	38	167	65
Total	262	370	325

^1^ Including sequences described as I-Jockey.

## Data Availability

The results of Blast2Go analyses are deposited in a Mendeley data folder “Contig sets for Georissa laseroni, Pleuropoma jana, Nerita melanotragus” [24]. Different contig numbers owing to re-assembly. The individual sequence reads are available as a GenBank Short Read Archive (SRA) BioProject (ID: PRJNA481126) with the run identifications: SRR7611487 https://dataview.ncbi.nlm.nih.gov/object/SRR7611487 (accessed on 4 June 2024) for *P. jana*. SRR7609217 https://dataview.ncbi.nlm.nih.gov/object/SRR7609217 (accessed on 4 June 2024) for *N. melanotragus*. SRR7522894 https://dataview.ncbi.nlm.nih.gov/object/SRR7522894 (accessed on 4 June 2024) for *G. laseroni*. The DNA contigs from the assemblies used in this manuscript are available in the GenBank WGS database submission SUB14449098 “Nerita, Pleuropoma, Georissa”.

## Supplementary Materials

The following supporting information can be downloaded at: https://www.mdpi.com/article/10.3390/genes15060783/s1, Supplementary information.docx including Supplementary Table S1: Details of next generation sequencing; Supplementary Figure S1: Maximum likelihood phylogeny of ORF2 in the Neritimorpha; Supplementary Figure S2: Maximum likelihood phylogeny of ORF2 in the Neritimorpha and other Gastropoda; alignments of the datasets used in this article (ORF1.fasta, ORF2_Gastropoda.fasta, ORF2_Neritimorpha.fasta).

## References

[1] Platt R.N., Vandewege M.W., Ray D.A. (2018). Mammalian transposable elements and their impacts on genome evolution. Chromosome Res..

[2] Böhne A., Brunet F., Galiana-Arnoux D., Schultheis C., Volff J.N. (2008). Transposable elements as drivers of genomic and biological diversity in vertebrates. Chromosome Res..

[3] Gilbert C., Peccoud J., Cordaux R. (2021). Transposable elements and the evolution of insects. Ann. Rev. Entomol..

[4] Sotero-Caio C.G., Platt R.N., Suh A., Ray D.A. (2017). Evolution and diversity of transposable elements in vertebrate genomes. Genome Biol. Evol..

[5] Shao F., Han M., Peng Z. (2019). Evolution and diversity of transposable elements in fish genomes. Sci. Rep..

[6] Pardos-Blas J.R., Irisarri I., Abalde S., Afonso C.M., Tenorio M.J., Zardoya R. (2021). The genome of the venomous snail *Lautoconus ventricosus* sheds light on the origin of conotoxin diversity. Gigascience.

[7] Song H., Li Z., Yang M., Shi P., Yu Z., Hu Z., Zhou C., Hu P., Zhang T. (2023). Chromosome-level genome assembly of the caenogastropod snail *Rapana venosa*. Sci. Data.

[8] Saenko S.V., Groenenberg D.S., Davison A., Schilthuizen M. (2021). The draft genome sequence of the grove snail *Cepaea nemoralis*. G3.

[9] Wicker T., Sabot F., Hua-Van A., Bennetzen J.L., Capy P., Chalhoub B., Flavell A., Leroy P., Morgante M., Panaud O.L. (2007). A unified classification system for eukaryotic transposable elements. Nat. Rev. Genet..

[10] Luchetti A., Šatović E., Mantovani B., Plohl M. (2016). RUDI, a short interspersed element of the V-SINE superfamily widespread in molluscan genomes. Mol. Genet. Genom..

[11] Matetovici I., Sajgo S., Ianc B., Ochis C., Bulzu P., Popescu O., Damert A. (2016). Mobile element evolution playing jigsaw—SINEs in gastropod and bivalve mollusks. Genome Biol. Evol..

[12] Piednoël M., Gonçalves I.R., Higuet D., Bonnivard E. (2011). Eukaryote DIRS1-like retrotransposons: An overview. BMC Genom..

[13] Thomas-Bulle C., Piednoël M., Donnart T., Filée J., Jollivet D., Bonnivard É. (2018). Mollusc genomes reveal variability in patterns of LTR-retrotransposons dynamics. BMC Genom..

[14] Raghavan N., Tettelin H., Miller A., Hostetler J., Tallon L., Knight M. (2007). Nimbus (BgI): An active non-LTR retrotransposon of the *Schistosoma mansoni* snail host *Biomphalaria glabrata*. Int. J. Parasitol..

[15] Han J.S. (2010). Non-long terminal repeat (non-LTR) retrotransposons: Mechanisms, recent developments, and unanswered questions. Mob. DNA.

[16] Khazina E., Weichenrieder O. (2009). Non-LTR retrotransposons encode noncanonical RRM domains in their first open reading frame. Proc. Nat. Acad. Sci. USA.

[17] Metcalfe C.J., Casane D. (2014). Modular organization and reticulate evolution of the ORF1 of Jockey superfamily transposable elements. Mob. DNA.

[18] Jurka J. (1998). Repeats in genomic DNA: Mining and meaning. Curr. Opin. Struct. Biol..

[19] Bao W., Kojima K.K., Kohany O. (2015). Repbase Update, a database of repetitive elements in eukaryotic genomes. Mob. DNA.

[20] Kapitonov V.V., Vladimir V., Tempel S., Jurka J. (2009). Simple and fast classification of non-LTR retrotransposons based on phylogeny of their RT domain potein sequences. Gene.

[21] Abrusán G., Krambeck H.J. (2006). Competition may determine the diversity of transposable elements. Theor. Popul. Biol..

[22] Bourque G., Burns K.H., Gehring M., Gorbunova V., Seluanov A., Hammell M., Imbeault M., Izsvák Z., Levin H.L., Macfarlan T.S. (2018). Ten things you should know about transposable elements. Genome Biol..

[23] Tambones I.L., Haudry A., Simão M.C., Carareto C.M. (2019). High frequency of horizontal transfer in Jockey families (LINE order) of drosophilids. Mob. DNA.

[24] Colgan D.J. (2024). Contig sets for *Georissa laseroni*, *Pleuropoma jana*, *Nerita melanotragus*. Mendeley Data V1. https://data.mendeley.com/datasets/vdj4rb9g9j/1.

[25] Uribe J.E., Colgan D.J., Castro L.R., Kano Y., Zardoya R. (2016). Phylogenetic relationships among superfamilies of Neritimorpha (Mollusca: Gastropoda). Mol. Phylogenet. Evol..

[26] Colgan D.J., Santos R.d.P. (2018). A phylogenetic classification of gastropod aquaporins. Mar. Genom..

[27] Conesa A., Götz S., Garcia-Gomez J.M., Terol J., Talon M., Robles M. (2005). Blast2GO: A universal tool for annotation, visualization and analysis in functional genomics research. Bioinformatics.

[28] Mizrokhi L.J., Georgieva S.G., Ilyin Y.V. (1988). Jockey, a mobile *Drosophila* element similar to mammalian LINEs, is transcribed from the internal promoter by RNA polymerase II. Cell.

[29] Priimagi A., Mizrokhi L., Ilyin Y. (1988). The *Drosophila* mobile element jockey belongs to LINEs and contains coding sequences homologous to some retroviral proteins. Gene.

[30] Lindberg D.R., Ponder W.F., Lindberg D.R. (2008). Patellogastropoda, Neritimorpha and Cocculinoidea: The low diversity gastropod clades. Phylogeny and Evolution of the Mollusca.

[31] Bandel K., Frýda J. (1999). Notes on the evolution and higher classification of the subclass Neritimorpha (Gastropoda) with the description of some new taxa. Geol. Palaeontol..

[32] Colgan D.J., Ponder W.F., Beacham E., Macaranas J.M. (2003). Gastropod phylogeny based on six segments from four genes representing coding or non-coding and mitochondrial or nuclear DNA. Molluscan Res..

[33] Kano Y., Chiba S., Kase T. (2002). Major adaptive radiation in neritopsine gastropods estimated from 28S rRNA sequences and fossil records. Proc. R. Soc. Lond. Ser. B Biol. Sci..

[34] Fuchs L.I.R., Knobloch J., Wiesenthal A.A., Fuss J., Franzenburg S., Torres Oliva M., Müller C., Wheat C.W., Hildebrandt J.P. (2023). A draft genome of the neritid snail *Theodoxus fluviatilis*. G3 Genes Genomes Genet..

[35] Afgan E., Sloggett C., Goonasekera N., Makunin I., Benson D., Crowe M., Gladman S., Kowsar Y., Pheasant M., Horst R. (2018). The Galaxy platform for accessible, reproducible and collaborative biomedical analyses: 2018 update. Nucleic Acids Res..

[36] Camacho C., Coulouris G., Avagyan V., Ma N., Papadopoulos J., Bealer K., Madden T.L. (2009). BLAST+: Architecture and applications. BMC Bioinform..

[37] Cock P.J.A., Chilton J.M., Grüning B., Johnson J.E., Soranzo N. (2015). NCBI BLAST+ integrated into Galaxy. Gigascience.

[38] Flynn J.M., Hubley R., Goubert C., Rosen J., Clark A.G., Feschotte C., Smit A.F. (2020). RepeatModeler2 for automated genomic discovery of transposable element families. Proc. Nat. Acad. Sci. USA.

[39] Rice P., Longden I., Bleasby A. (2000). EMBOSS: The European Molecular Biology Open Software Suite. Trends Genet..

[40] Cock P.J.A., Grüning B.A., Paszkiewicz K., Pritchard L. (2013). Galaxy tools and workflows for sequence analysis with applications in molecular plant pathology. PeerJ.

[41] Shen W., Le S., Li Y., Hu F. (2016). SeqKit: A cross-platform and ultrafast toolkit for FASTA/Q file manipulation. PLoS ONE.

[42] Hall T.A. (1999). BioEdit: A user–friendly biological sequence alignment editor and analysis program for Windows 95/98/NT. Nucleic Acids Res. Symp. Ser..

[43] Larkin M.A., Blackshields G., Brown N.P., Chenna R., McGettigan P.A., McWilliam H., Valentin F., Wallace I.M., Wilm A., Lopez R. (2007). Clustal W and Clustal X version 2.0. Bioinformatics.

[44] Miller M.A., Pfeiffer W., Schwartz T. Creating the CIPRES Science Gateway for inference of large phylogenetic trees. Proceedings of the Gateway Computing Environments Workshop (GCE).

[45] Stamatakis A., Hoover P., Rougemont J. (2008). A rapid bootstrap algorithm for the RAxML web-servers. Syst. Biol..

[46] Pattengale N.D., Alipour M., Bininda-Emonds O.R.P., Moret B.M.E., Stamatakis A. (2010). How many bootstrap replicates are necessary?. J. Comp. Biol..

[47] Le S.Q., Gascuel O. (2008). An improved general amino acid replacement matrix. Mol. Biol. Evol..

[48] Rambaut A. Figtree v. 1.4.2. http://tree.bio.ed.ac.uk/software/figtree/.

[49] Tamura K., Stecher G., Kumar S. (2021). MEGA11: Molecular evolutionary genetics analysis version 11. Mol. Biol. Evol..

[50] Adachi K., Yoshizumi A., Kuramochi T., Kado R., Okumura S.I. (2021). Novel insights into the evolution of genome size and AT content in mollusks. Mar. Biol..

[51] Pimentel S.C., Upton H.E., Collins K. (2022). Separable structural requirements for cDNA synthesis, nontemplated extension, and template jumping by a non-LTR retroelement reverse transcriptase. J. Biol. Chem..

[52] Fernández-Medina R.D., Ribeiro J.M., Carareto C.M., Velasque L., Struchiner C.J. (2012). Losing identity: Structural diversity of transposable elements belonging to different classes in the genome of *Anopheles gambiae*. BMC Genom..

[53] Luchetti A., Mantovani B. (2013). Non-LTR R2 element evolutionary patterns: Phylogenetic incongruences, rapid radiation and the maintenance of multiple lineages. PLoS ONE.

