# Supplementary material for: The Families of Non-LTR Transposable Elements within Neritimorpha and Other Gastropoda
Source: Genes (Basel). 2024 Jun 14;15(6):783. doi: 10.3390/genes15060783 (PMC11203168; doi:10.3390/genes15060783)
Supplement: Supplementary file 1 [file genes-15-00783-s001.zip › Supplementary Figure S2.pdf]

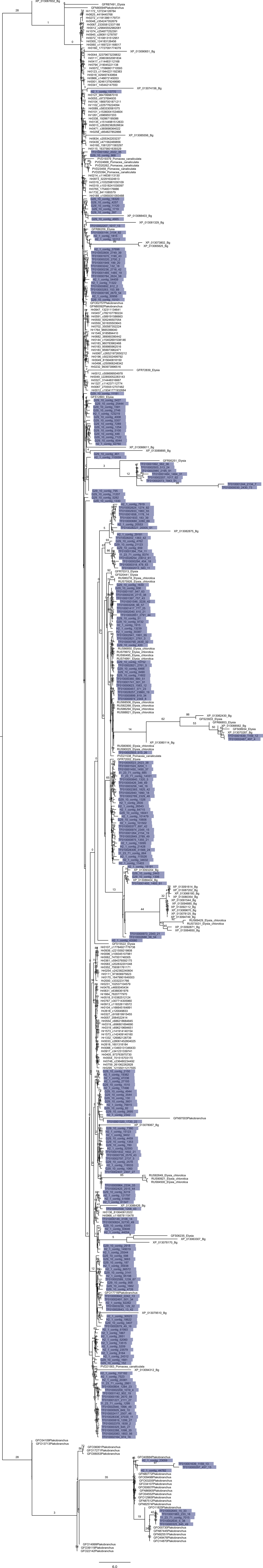
6.0
