# Supplementary material for: The Families of Non-LTR Transposable Elements within Neritimorpha and Other Gastropoda
Source: Genes (Basel). 2024 Jun 14;15(6):783. doi: 10.3390/genes15060783 (PMC11203168; doi:10.3390/genes15060783)
Supplement: Supplementary file 1 [file genes-15-00783-s001.zip › Supplementary Information.pdf]

**Supplementary Information**

The protein sequence alignment files in the Supplementary information are those used for Figures 1 and 2, and the Supplementary Figures S1 and S2. For GenBank data. the source of individual sequences is generally  indicated by accession numbers and species name (sometimes truncated). Some sequences were extracted from the GenBank WGS. Those from *Theodoxus fluviatilis* are indicated by scaffold number in the draft genome followed by the contig number and the relevant ORF from the contig. Sequences with the initial letters QXJH are from *Haliotis rubra* isolate DU_JTF1 Scf966, whole genome shotgun sequence (these letters are replaced by "Hr" in Supplementary Figure S1). The source for sequences from the genomic skimming surveys is indicated by Gl for *Georissa laseroin*, Pj for *Pleuropoma jana* or Nm for *Neritan melanotragus.*  These sequences are identified by contig numbers followed by the ORF number from the contig.

**Supplementary Figure S1. Maximum likelihood phylogeny of ORF2 in the Neritimorpha.**

This is the complete topology for Figure 2 in the main text, showing the identification of all sequences included in the analysis.

**Supplementary Figure S2. Maximum likelihood phylogeny of ORF2 in the Neritimorpha and other Gastropoda.**

TEs in other gastropods similar to those found here were investigated by using representatives from diverse neritimorph clades (the "classification sequences" ) to the GenBank non-redundant protein database using blastn, limiting the search to Gastropoda. These sequences were downloaded and filtered to remove duplicates and those less than 230 amino acids long. These searches did not find any vetigastropod sequences so they were extended to tblastn queries of *Haliotis rubra* genomic survey sequences using *P. jana* contigs 4459 1581,1719, 82 and 2443 (from disparate neritimorph clades). The *H. rubra* data were filtered as for other sequences. The filtered GenBank sequences were combined with the neritimorph dataset and re-aligned with ClustalX2. The combined alignment was reduced by removing GenBank sequences with E values of more than E-10 in blast searches with AAA28675, neritimorph sequences with E values more than E-10 (when compared to the original conspecific search sequence) and sequences of less than 430 positions. The dataset was then re-aligned prior to phylogenetic analysis by RAxML using the procedure detailed in the main text.

For GenBank data. the source of individual sequences is indicated by accession numbers and species name (truncated) or the scaffold number in the *T. fluviatilis genome* followed by the contig number and the relevant ORF from the contig. The source for sequences from the genomic skimming surveys

is indicated by Gl for *Georissa laseroin*, Pj for *Pleuropoma jana* or Nm for *Neritan melanotragus.* These sequences are identified by contig numbers. The topology is rooted on a clade containing the classification sequence Nm contig 7015 which belongs to the Nimbus TE family. Bootstrap values are shown along branches. Sequences from the Neritimorpha are highlighted with blue. The scale bar represents estimated substitutions per site.

**Supplementary Table 1.** Details of next generation sequencing. The rows in order show the numbers of base pairs for which 50 and 25 percent of the contigs have at least the specified length, the maximum contig lengths, the total number of contigs and the number of bases included in the contig set.

| **Species** **Measure** | ***Nerita melanotragus*** | ***Georissa laseroni*** | ***Pleuropoma jana*** |
|---|---|---|---|
| **Contigs** | | | |
| N50 | 325 | 496 | 273 |
| N25 | 498 | 1,400 | 356 |
| Maximum | 15,817 | 17,860 | 15,889 |
| Number of contigs | 1,736,934 | 597,696 | 1,322,892 |
| **Summed contig lengths** | 555,439,204 | 245,206,592 | 362,665,078 |
